# *AGER* rs2070600 polymorphism elevates neutrophil-lymphocyte ratio and mortality in metastatic lung adenocarcinoma

**DOI:** 10.18632/oncotarget.21764

**Published:** 2017-10-10

**Authors:** Kakuhiro Yamaguchi, Hiroshi Iwamoto, Shinjiro Sakamoto, Yasushi Horimasu, Takeshi Masuda, Shintaro Miyamoto, Taku Nakashima, Shinichiro Ohshimo, Kazunori Fujitaka, Hironobu Hamada, Noboru Hattori

**Affiliations:** ^1^ Department of Molecular and Internal Medicine, Graduate School of Biomedical and Health Sciences, Hiroshima University, Hiroshima, Japan; ^2^ Department of Emergency and Critical Care Medicine, Graduate School of Biomedical Sciences, Hiroshima University, Hiroshima, Japan; ^3^ Department of Physical Analysis and Therapeutic Sciences, Graduate School of Biomedical and Health Sciences, Hiroshima University, Hiroshima, Japan

**Keywords:** *AGER* polymorphism, neutrophil-lymphocyte ratio, lung cancer, metastatic adenocarcinoma, disease susceptibility

## Abstract

**Background:**

The receptor for advanced glycation end-product (RAGE) is a multi-ligand receptor involved in inflammation. In the gene encoding RAGE (*AGER*), there are three well-known polymorphisms; rs2070600, rs1800624, and rs1800625, which potentially increase the risk of lung cancer. Remarkably, *AGER* rs2070600 polymorphism, which increases ligand-binding affinity, is a potential prognostic factor in non-small cell lung cancer, but the underlying mechanism is unclear. The neutrophil-lymphocyte ratio (NLR) reflects tumor-associated systemic inflammatory conditions; high ratios are associated with poor prognosis in multiple cancers. Additionally, some humoral factors via RAGE-signaling are associated with elevated NLR in cancer patients.

**Objectives:**

Associations of *AGER* polymorphisms with disease susceptibility, prognosis, and NLR were investigated in Japanese patients with lung adenocarcinoma.

**Methods:**

We included 189 patients with lung adenocarcinoma, 96 of which had distant metastases, and 303 healthy controls. The correlation between *AGER* polymorphisms (rs2070600, rs1800624, rs1800625) and disease susceptibility and factors elevating the mortality and NLR in patients with metastases were evaluated.

**Results:**

Only the minor allele of rs2070600 was associated with a higher NLR (*β* = 0.209, *p* = 0.043) and a poor prognosis (Hazard ratio = 2.06, 95% Confidence interval = 1.09–3.77, *p* = 0.028) in patients with metastatic disease, independently of background characteristics, including EGFR mutation status. All three polymorphisms were not associated with the risk of lung adenocarcinoma.

**Conclusions:**

The *AGER* rs2070600 polymorphism was independently associated with systemic inflammation and poor prognosis in patients with metastatic lung adenocarcinoma.

## INTRODUCTION

Lung cancer is the leading cause of cancer deaths worldwide; the most common form of lung cancer is lung adenocarcinoma in both non-smokers and smokers [1]. In patients with lung adenocarcinoma, the epidermal growth factor receptor (EGFR) mutation status is an important prognostic factor. The presence of EGFR mutations is associated with a favorable prognosis, especially when patients are treated with EGFR tyrosine kinase inhibitors (TKIs) [2–4]. Recent work has also demonstrated that tumor-associated systemic inflammatory conditions might be a prognostic factor in cancer patients [5, 6]. An elevated neutrophil-lymphocyte ratio (NLR), an indicator of the systemic inflammatory response, is associated with shorter survival in patients with lung cancer as well as other cancers [7–9].

The receptor for advanced glycation end-product (RAGE) is a transmembrane receptor that can bind to numerous ligands, such as high mobility group box-1, and RAGE/ligand interactions initiate pro-inflammatory intracellular signaling pathways [10–12]. Previous studies have shown that post-RAGE signaling promotes the production of several growth factors and cytokines, e.g., platelet-derived growth factor (PDGF), interleukin-6 (IL-6), and monocyte chemotactic protein-1 (MCP-1) [13], which might be associated with aggravating tumor-related systemic inflammation and elevated NLRs [14, 15].

The gene encoding RAGE (*AGER*) contains functional polymorphisms; rs2070600, rs1800624, and rs1800625. rs2070600 polymorphism, which is located at a ligand-binding site in exon 3, increases the ligand-binding affinity of RAGE and enhances post-RAGE signaling [16–18]. rs1800624 and rs1800625 polymorphisms, which are located promoter region, influences the transcriptional activity of RAGE promoter, although it remains controversial whether these polymorphisms act positively or negatively [19–22]. These three polymorphisms were reported as the risk of lung cancer and breast cancer [23, 24], but conflicting results have been obtained with respect to the association between the rs2070600 polymorphism and lung cancer risk. Young et al. found no association between rs2070600 polymorphism and susceptibility to lung cancer in the Caucasian population [25], but two reports have shown that this polymorphism is associated with lung cancer susceptibility in the Han Chinese population [26, 27]. Additionally, Wang et al. have shown that the *AGER* rs2070600 minor allele is associated with a poorer prognosis in non-small cell lung cancer (NSCLC) patients, although the EGFR mutation status was not included in the survival analysis and the underlying mechanisms are not known [26].

We hypothesized that the *AGER* polymorphisms aggravate tumor-associated systemic inflammatory conditions, as indicated by elevated NLRs, and therefore *AGER* polymorphism could predict survival in patients with lung adenocarcinoma, independently of the effect of EGFR mutations. In this study, we initially examined the correlation between *AGER* polymorphisms and disease susceptibility for lung adenocarcinoma in the Japanese population. We subsequently investigated the factors elevating mortality and NLR in patients with metastatic lung adenocarcinoma, including the effects of *AGER* polymorphisms and EGFR mutation status.

## RESULTS

### Subject characteristics

The patients with lung adenocarcinoma were significantly older (64.3 ± 11.0 and 55.5 ± 7.8, *p* < 0.001), more male-dominant (113/189 and 247/303, *p* < 0.001), and had higher pack-year smoking histories (26.6 ± 28.5 and 18.0 ± 21.9, *p* = 0.002) than those of controls (Table 1). The genotype frequencies for the *AGER* polymorphisms did not deviate from Hardy-Weinberg equilibrium (*p* > 0.05).

**Table 1:** Baseline characteristics

Variable	Patients	Controls	*p*-value
Subjects	189	303	
Age, years	64.3 ± 11.0	55.5 ± 7.8	<0.001^**^
Sex, male/female	113/76	247/56	<0.001^**^
Smoking history, pack years	26.6 ± 28.5	18.0 ± 21.9	0.002^*^
EGFR mutations, +/-/unknown	43/133/13		
Stage, I/II/III/IV	38/9/46/96	-	

^*^: *p* < 0.05, ^**^: *p* < 0.001 Mann–Whitney *U*-test.

Values are means ± SD, unless stated otherwise.

EGFR, epidermal growth factor receptor.

### AGER polymorphisms and the risk of lung adenocarcinoma

The genotype distributions of *AGER* polymorphisms (rs2070600, rs1800624, rs1800625) showed no significant difference between patients with lung adenocarcinoma and healthy controls in the Japanese population (Table 2). Subset analysis also revealed that no significant difference in genotype distributions of the *AGER* polymorphisms was found between the patients with EGFR mutation positive/negative lung adenocarcinoma and healthy controls (Table 2).

**Table 2:** Associations of the *AGER* polymorphisms with the risk of lung adenocarcinoma

	rs2070600 genotype distribution			*p*-value^*^	rs1800624 genotype distribution			*p*-value^*^	rs1800625 genotype distribution			*p*-value^*^
	C/C (%)	C/T (%)	T/T (%)		C/C	C/T	T/T		A/A	A/G	G/G	
All patients^†^ (n = 189)	144	42	3	0.671	112	63	14	0.690	160	24	5	0.652
	(76.2)	(22.2)	(1.6)		(59.3)	(33.3)	(7.4)		(84.7)	(12.7)	(2.6)	
patients with EGFR mutation-positive (n = 43)	32	11	0	0.645	27	13	3	0.634	39	4	0	0.666
	(74.4)	(25.6)	(0.0)		(62.8)	(30.2)	(7.0)		(90.7)	(9.3)	(0.0)	
patients with EGFR mutation-negative (n = 133)	100	30	3	0.771	78	46	9	0.855	112	16	5	0.317
	(75.2)	(22.6)	(2.2)		(58.6)	(34.6)	(6.8)		(84.2)	(12.0)	(3.8)	
Controls (n = 303)	219	78	6		169	113	21		254	44	5	
	(72.3)	(25.7)	(2.0)		(55.8)	(37.3)	(6.9)		(83.8)	(14.5)	(1.7)	

^*^: Fisher’s exact test, compared with control subjects.

^†^: All patients included 13 patients whose EGFR mutation status had been unknown.

*AGER*, gene encoding the receptor for advanced glycation end-product; CI, confidence interval; EGFR, epidermal growth factor receptor.

### Association of the AGER rs2070600 polymorphism with NLR in patients with metastatic disease

The 96 patients with metastatic lung adenocarcinoma included 71 patients with the C/C genotype and 25 patients with the C/T and T/T genotypes of rs2070600 polymorphism (Table 3). The frequency of patients with a performance status (PS) of greater than or equal to 2 was significantly lower in patients with the C/C genotype than in those with the C/T and T/T genotypes (12/71 and 9/25, *p* = 0.047).

**Table 3:** Baseline characteristics in the patients with metastatic lung adenocarcinoma

Variables	rs2070600 C/C genotype	rs2070600 C/T&T/T genotype	*p*-value
Subjects	71	25	
Age, years	61.2 ± 11.5	63.2 ± 10.8	0.523
Sex, male/female	38/33	10/15	0.245
Smoking history, pack years	23.4 ± 32.0	18.8 ± 24.3	0.815
PS, 0–1/≥2	59/12	16/9	0.047^*^
HbA1c, %	6.0 ± 0.7	6.3 ± 0.9	0.081
EGFR mutations, +/-	28/43	9/16	0.761
Platinum-based chemotherapy, +/-	48/23	12/13	0.082
T category of TNM classification, 1/2/3/4/X	10/28/4/28/1	1/6/2/16/0	0.142
N category of TNM classification, 0/1/2/3	12/5/23/31	5/1/5/14	0.583

^*^: *p* < 0.05, Pearson’s Chi-squared test.

Values are mean ± SD unless stated otherwise.

EGFR, epidermal growth factor receptor; PS, performance status.

**Figure 1:**
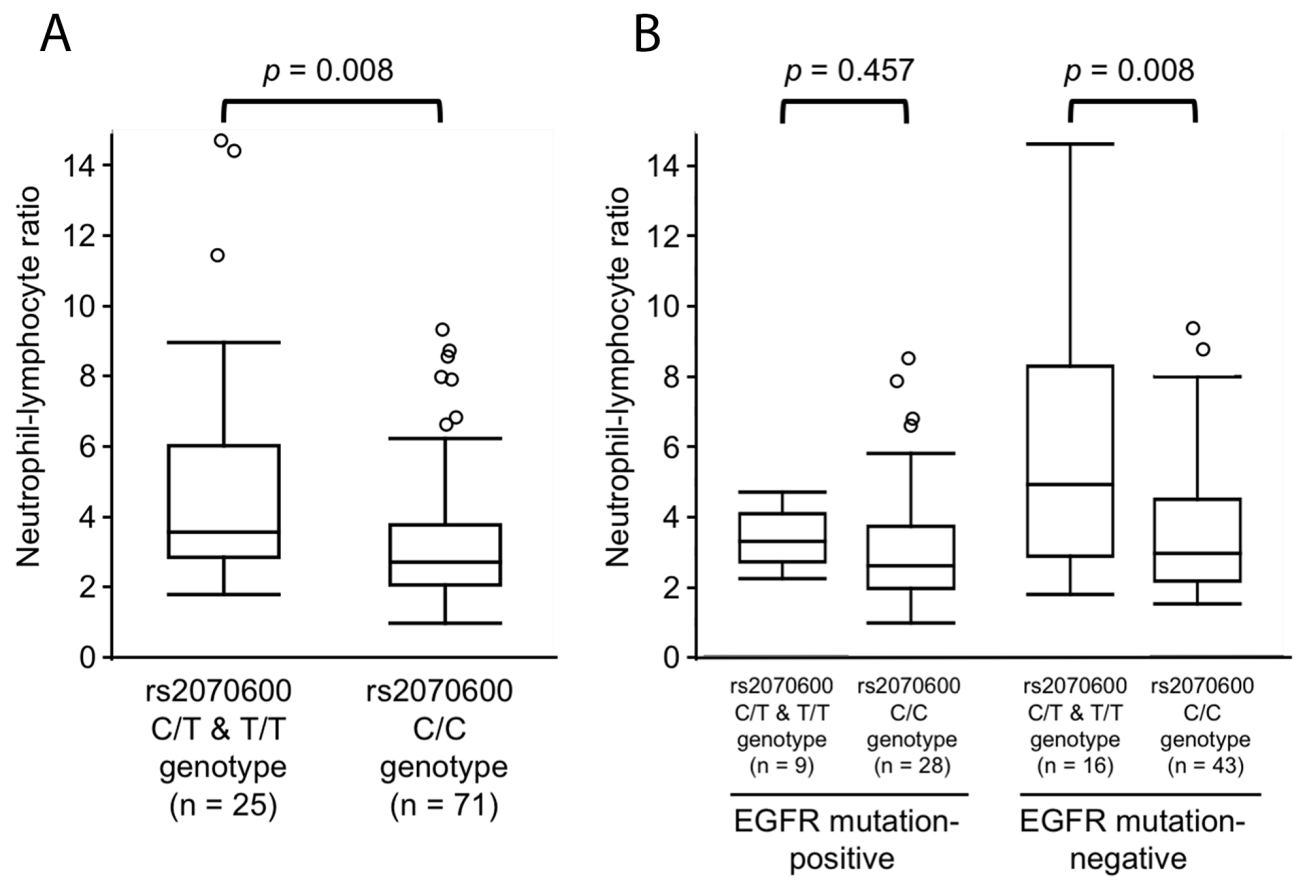
Box plots showing the range of neutrophil-lymphocyte ratios with/without the *AGER* rs2070600 minor allele (T) in the patients with metastatic lung adenocarcinoma (n = 96) **(A),** and exploratory subgroup analysis in patients with EGFR mutation-positive and EGFR mutation-negative lung adenocarcinoma, separately **(B).** Each box represents the 25^th^ to 75^th^ percentiles; solid lines within the boxes indicate the median values; whiskers are the 10^th^ and 90^th^ percentiles; and points represent outliers. The *p*-values were evaluated using Mann–Whitney *U*-tests.

The NLR in patients with the *AGER* rs2070600 minor allele (T) was significantly higher than that in patients without the minor allele (5.08 ± 3.61 and 3.38 ± 1.94, *p* = 0.008) (Figure 1A). In exploratory subgroup analysis, there was a significant difference in NLR between the EGFR mutation-negative patients with/without rs2070600 minor allele, but not between the EGFR mutation-positive patients with/without rs2070600 minor allele (Figure 1B). A univariate linear regression analysis revealed that the NLR was significantly associated with PS (≥2), the T category in the TNM classification, and the presence of the rs2070600 minor allele (T) (*t* = 2.16, *β* = 0.218, *p* = 0.033; *t* = 2.53, *β* = 0.254, *p* = 0.013; *t* = 2.96, *β* = 0.292, *p* = 0.004, respectively). In a multivariate stepwise linear regression analysis, the rs2070600 minor allele (T) was independently correlated with a higher NLR (*t* = 2.06, *β* = 0.209, *p* = 0.043) (Table 4).

**Table 4:** Correlation between NLR and baseline characteristics (*n* = 96)

Variables	*t*	*β*	*p*-value
Univariate analysis			
Age, years	-0.04	-0.004	0.967
Sex, male	-0.70	-0.072	0.484
Smoking history, pack years	-1.15	-0.119	0.252
PS, ≥2/0–1	2.16	0.218	0.033^*^
HbA1c, %	0.21	0.022	0.833
EGFR mutations, +/−	-0.82	-0.084	0.417
T category of TNM classification	2.53	0.254	0.013^*^
N category of TNM classification	-0.40	-0.042	0.687
rs2070600 minor allele (T), +/−	2.96	0.292	0.004^*^
rs1800624 minor allele (T), +/−	0.53	0.055	0.596
rs1800625 minor allele (G), +/−	1.37	0.140	0.174
			
Multivariate stepwise analysis^†^			
PS, ≥2/0–1	1.68	0.167	0.097
T category of TNM classification	1.83	0.183	0.070
Rs2070600 minor allele (T), +/−	2.06	0.209	0.043^*^

^*^: *p* < 0.05 Linear regression analysis.

^†^: Other independent variables included in the model were all variables in the univariate analysis.

EGFR, epidermal growth factor receptor; NLR, neutrophil-lymphocyte ratio; PS, performance status.

On the other hand, no significant association with NLR was found for rs1800624 and rs1800625 polymorphisms (rs1800624 A/A and A/T+T/T: 3.94 ± 2.83 and 3.66 ± 2.19, *p* = 0.964; rs1800625 A/A and A/G+G/G: 3.95 ± 2.66 and 2.77 ± 1.32, *p* = 0.108, respectively) (Table not shown).

### Confirmation of the prognostic value of NLR in patients with metastatic disease

**Figure 2:**
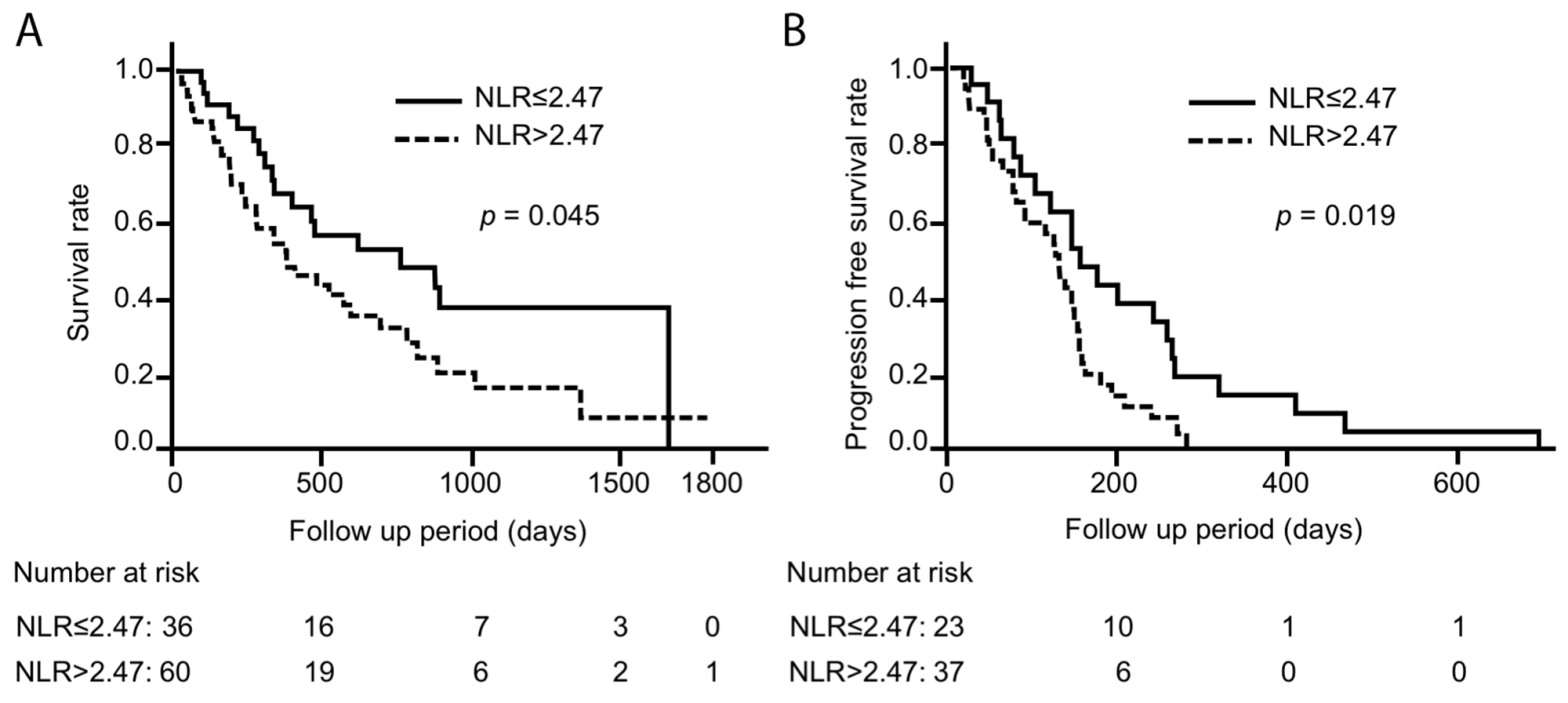
Kaplan-Meier analysis showing **(A)** the survival rate and **(B)** progression free survival (PFS) with platinum-based chemotherapy in patients with metastatic lung adenocarcinoma, based on above or below 2. 47 of NLR (n = 96). Solid line indicates patients with lower NLR. Dotted line indicates patients with higher NLR. Patients with higher NLR showed significantly poorer survival and shorter PFS compared to those with lower NLR.

The optimal cut-off levels of NLR for predicting 5-year survival rates was 2.47, which was determined by receiver operating characteristic (ROC) curve analysis. The patients with higher NLR showed a significant shorter survival than those with lower NLR (median survival time; 400 days and 787 days, *p* = 0.045) (n = 96) (Figure 2A). Additionally, progression free survival (PFS) with platinum-based chemotherapy was significantly shorter in the patients with higher NLR than those with lower NLR (median; 133 days and 158 days, *p* = 0.019) (n = 60) (Figure 2B). A univariate Cox proportional hazards analysis revealed that a higher NLR was significantly associated with a poor prognosis (HR = 1.16, 95%CI = 1.04–1.28, *p* = 0.008). In the multivariate analysis, a higher NLR was also a significant predictor of 5-year mortality, independent of age, sex, smoking history, PS, EGFR mutation status, platinum-based chemotherapy, and the N category of the TNM classification (HR = 1.17, 95%CI = 1.03–1.30, *p* = 0.016) (Table 5).

**Table 5:** Predictive value for 5-year mortality in patients with metastatic lung adenocarcinoma assessed by Cox proportional hazards model (*n* = 96)

Variable	Univariate analysis			Multivariate analysis					
				model 1 (NLR)			model 2 (rs2070600 polymorphism)		
	HR	95%CI	*p*-value	HR	95%CI	*p*-value	HR	95%CI	*p*-value
Age, years	0.99	0.97-1.02	0.567	1.00	0.97-1.03	0.790	1.00	0.97-1.02	0.730
Sex, male	1.79	1.05-3.06	0.031^*^	1.17	0.58-2.41	0.661	1.14	0.50-2.07	0.952
Smoking history, pack years	1.01	1.00-1.01	0.032^*^	1.01	0.99-1.02	0.175	1.02	0.99-1.02	0.285
PS, ≥2	2.69	1.39-4.93	0.004^*^	2.88	1.37-5.77	0.006^*^	3.33	1.59-6.66	0.002^*^
HbA1c, %	1.14	0.77-1.60	0.486						
EGFR mutations, +	0.41	0.22-0.72	0.002^*^	0.27	0.13-0.53	< 0.001^*^	0.27	0.13-0.52	< 0.001^*^
Platinum-based chemotherapy, +	0.46	0.27-0.81	0.007^*^	0.36	0.19-0.70	0.003^*^	0.42	0.21-0.82	0.012^*^
T category	1.17	0.92-1.50	0.195						
N category	1.31	1.01-1.74	0.040^*^	1.47	1.11-2.02	0.007^*^	1.43	1.08-1.96	0.012^*^
NLR	1.16	1.04-1.28	0.008^*^	1.17	1.03-1.30	0.016^*^			
rs2070600 minor allele (T), +	2.08	1.14-3.65	0.019^*^				2.06	1.09-3.77	0.028^*^
rs1800624 minor allele (T), +	0.75	0.43-1.28	0.295						
rs1800625 minor allele (T), +	0.75	0.31-1.55	0.455						

^*^: *p* < 0.05 Cox proportional hazards model.

EGFR, epidermal growth factor receptor; NLR, neutrophil-lymphocyte ratio; PS, performance status.

### AGER rs2070600 polymorphism elevated mortality in patients with metastatic disease

**Figure 3:**
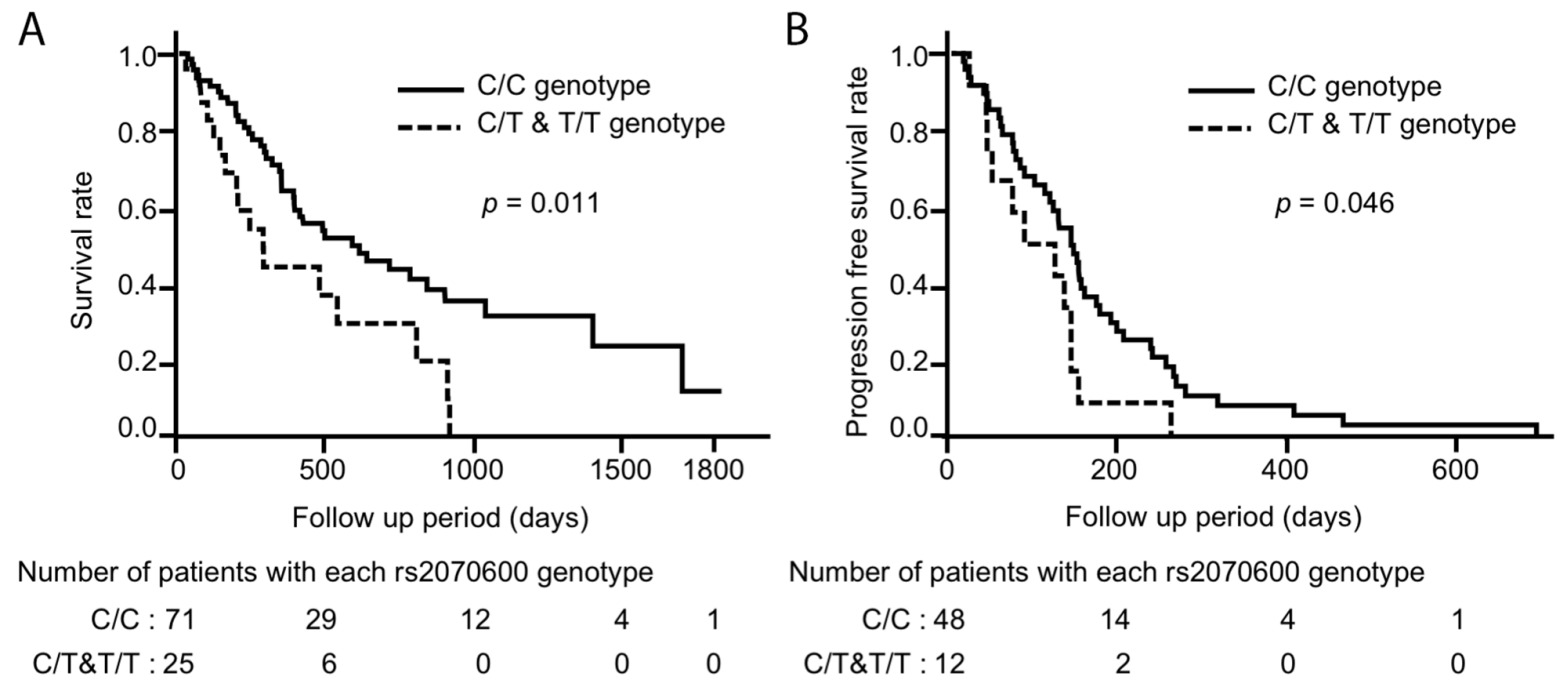
Kaplan-Meier analysis showing **(A)** the survival rate and **(B)** progression free survival (PFS) with platinum-based chemotherapy in patients with metastatic lung adenocarcinoma, on the presence or absence of the *AGER* rs2070600 minor allele (T) (n = 96). Solid line indicates patients with the C/C genotype. Dotted line indicates patients with the C/T or T/T genotype. Patients with the *AGER* rs2070600 minor allele (T) showed significantly poorer survival and shorter PFS compared to those without the minor allele.

The 5-year survival rates were significantly lower in patients with the *AGER* rs2070600 minor allele (T) than in those without this allele (median survival time; 294 days and 593 days, *p* = 0.011) (n = 96) (Figure 3A). Additionally, PFS with platinum-based chemotherapy was significantly shorter in the patients with rs2070600 minor allele (T) than those without (median; 111 days and 148 days, *p*=0.046) (n = 60) (Figure 3B). In a univariate Cox proportional hazards analysis, the presence of the rs2070600 minor allele (T) was significantly correlated with a poor prognosis (HR = 2.08, 95%CI = 1.14–3.65, *p* = 0.019). In a multivariate Cox proportional hazards analysis, the presence of the rs2070600 minor allele (T) was an independent predictor of 5-year mortality adjusted for age, sex, smoking history, PS, EGFR mutation status, platinum-based chemotherapy, and the N category of the TNM classification (HR = 2.06, 95%CI = 1.09–3.77, *p* = 0.028) (Table 5).

However, there was no significant association of the 5-year survival rates with rs1800624 polymorphism and rs1800625 polymorphisms in patients with metastatic lung adenocarcinoma (Table 5).

## DISCUSSION

In the present study, we showed that the *AGER* rs2070600 minor allele was an independent predictor of elevated NLR and poor survival in patients with metastatic lung adenocarcinoma. We also confirmed that a higher NLR was associated with a poor prognosis in these patients. These results suggest that the rs2070600 minor allele may enhance tumor-associated systemic inflammation, which is related to a poor prognosis in these patients. Additionally, the rs2070600 minor allele was significantly correlated with a poor survival in patients with metastatic lung adenocarcinoma, independently of EGFR mutations status, indicating that this polymorphism could be a useful prognostic marker for this group of patients. However, *AGER* polymorphisms; rs2070600, rs1800624, and rs1800625, did not increase the risk of lung adenocarcinoma in the Japanese population.

We demonstrated that the *AGER* rs2070600 minor allele was independently associated with augmented systemic inflammation, as evidenced by a higher NLR, which may have a negative impact on survival and response to platinum-based chemotherapy in these patients. Although the mechanism of the association between the rs2070600 minor allele and a higher NLR is still unclear, previous reports have shown that the rs2070600 minor allele variant enhances the ligand-binding affinity of RAGE and post-RAGE signaling, which induces the expression of pro-inflammatory and/or cell-proliferating factors, e.g., PDGF, IL-6, and MCP-1 [13, 17, 18]. Additionally, higher levels of these humoral factors in circulation are significantly associated with elevated NLR in cancer patients [15]. Based on these observations, we hypothesize that augmented post-RAGE signaling by the rs2070600 polymorphism may aggravate systemic inflammation and elevate the NLR in patients with metastatic lung adenocarcinoma. It should be noted that RAGE expression in the tumor is lower than that in normal lung tissues [28, 29] and is not associated with survival in patients with lung cancer [30]. However, tumor-associated macrophages (TAM) exhibit high expression of RAGE, and post-RAGE signaling in TAM contributes to tumor progression by deteriorating the inflammatory tumor microenvironment [31]. On the other hand, there was no significant difference of NLR by rs2070600 polymorphism in EGFR-positive patients in the exploratory subgroup analysis, which, if confirmed, may suggest a smaller effect of this polymorphism on inflammatory response in EGFR mutation-positive disease. Further studies are needed to elucidate the mechanism by which the rs2070600 polymorphism aggravates systemic inflammatory conditions.

In the present study, the *AGER* rs2070600 polymorphism was significantly associated with a poor prognosis in Japanese patients with lung adenocarcinoma, although the risk of the disease was not associated with all three *AGER* polymorphisms; rs2070600, rs1800624, and rs1800625. Wang et al. showed that this polymorphism is also associated with a poor prognosis in Han Chinese patients with NSCLC [26], suggesting that the prognostic value of this polymorphism is reproducible among different ethnicities. However, the association between the *AGER* polymorphisms and the risk of lung cancer might depend on ethnicity. Especially about rs2070600 polymorphism, conflicting results have been obtained; two reports have shown an association between the rs2070600 polymorphism and the risk of lung cancer in the Han Chinese population [26, 27], but no association was detected between the same polymorphism and the risk of lung cancer in the Caucasian population [25], in agreement with the present results for Japanese patients with lung adenocarcinoma. One potential explanation for these contradictory results is ethic differences in the allele frequencies of the rs2070600 polymorphism; the minor allele frequency of the rs2070600 polymorphism in the Han Chinese population was reported to be 29%, which is much higher than 6% in the Caucasian population and 13% in the Japanese population [32]. The differences among studies may also be explained by differences in lung cancer histology; our cohort was limited to patients with lung adenocarcinoma, whereas over half of the patients did not have lung adenocarcinoma in previous studies showing that rs2070600 polymorphism is associated with lung cancer susceptibility [26, 27]. Therefore, further studies considering histological subtype and/or ethnicity are needed to verify our results.

The present study had several limitations. First, the inclusion period was relatively long and the sample size was relatively small. There were also significant differences in baseline characteristics between cancer patients and healthy controls. We additionally checked the correlation between *AGER* polymorphisms and disease susceptibility after adjusting for sex and smoking history, and did not find an increased risk of lung adenocarcinoma (data not shown). Second, the echinoderm microtubule-associated protein-like 4-anaplastic lymphoma kinase (*EML4-ALK*) fusion gene was not evaluated in this study because most patients were diagnosed before using ALK-TKI. In general, the frequency of the *EML4-ALK* fusion gene in lung adenocarcinoma is relatively low compared with that of EGFR mutations in the Japanese population (6.7% and 45%, respectively) [33, 34]. Therefore, the *EML4-ALK* fusion gene likely did not affect the study results. Lastly, further investigations are needed to elucidate the clinical impact of rs2070600 polymorphism in patients with metastatic lung adenocarcinoma. One reason is that the prognostic value of this polymorphism is not statistically superior to that of NLR (data not shown), although genetic marker is less readily available compared to NLR which can be obtained in routine blood test. However, in contrast with rs2070600 polymorphism, NLR is a continuous variables and its optimal cut-off levels are still unclear [7], and could be changed by acquired factors, e.g. infection, corticosteroids therapy, and diabetes. Another reason is that RAGE signaling in TAM contribute to increasing production of vascular endothelial growth factor (VEGF), which is also increased by rs2070600 minor allele [18, 31]. These data let us speculate that patients with metastatic lung adenocarcinoma having this polymorphism potentially get more clinical benefit of anti-VEGF therapy, such as bevacizumab, although we could not evaluate this point because this study contained only 7 patients who were received bevacizumab administration.

In conclusion, the present results suggest that the *AGER* rs2070600 polymorphism in patients with metastatic lung adenocarcinoma is associated with systemic inflammatory conditions, as indicated by higher levels of NLR, which may have a negative effect on survival of patients harboring this polymorphism. We also found that the *AGER* rs2070600 polymorphism is associated with a poorer prognosis for patients with metastatic lung adenocarcinoma, independent of the EGFR mutation status. Although further studies are warranted to clarify the causal relationship, our results suggest that the *AGER* rs2070600 polymorphism is a potential genetic marker for tumor-related inflammatory conditions and a poor prognosis in patients with metastatic lung adenocarcinoma.

## MATERIALS AND METHODS

### Subjects

**Figure 4:**
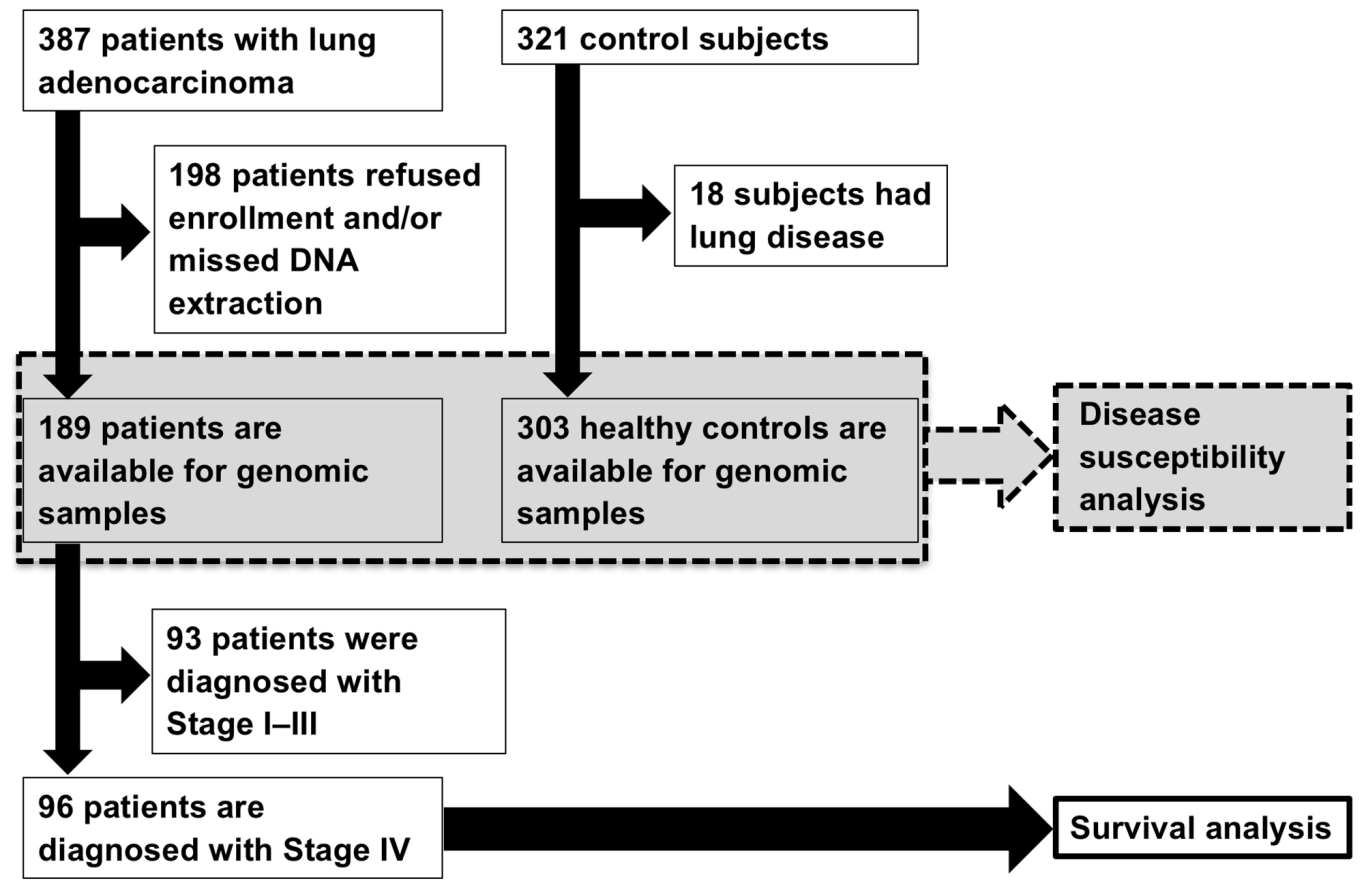
Flow chart diagram of subject selection One hundred eighty-nine patients with lung adenocarcinoma and 303 healthy controls were enrolled in the analysis of disease susceptibility. In the 96 patients with metastatic lung adenocarcinoma, the associations of rs2070600 polymorphism with mortality and neutrophil-lymphocyte ratio were evaluated.

We collected blood samples from 387 Japanese patients with lung adenocarcinoma, who were diagnosed by pathology and/or cytology at Hiroshima University Hospital (Hiroshima, Japan) between April 2002 and March 2012. We also gathered blood samples and medical check-up data from 321 volunteers. The inclusion criteria were shown in Figure 4. Finally, we enrolled 189 patients with lung adenocarcinoma, classified as Stage I–IV, and 303 healthy controls in the present analysis for evaluating the association of *AGER* polymorphisms with the disease susceptibility of lung adenocarcinoma (Figure 4). The cancer stage was determined based on the seventh edition of the TNM classification [35]. Ninety-six of 189 patients, classified as Stage IV based on the presence of distant metastatic sites, were enrolled in survival analysis. All the patients with metastatic disease had not been treated with chemotherapy and/or radiotherapy, and no patient took oral corticosteroid at their first visit. Sixty-three percent of patients with metastatic disease were subsequently treated with platinum-based chemotherapy, which included cisplatin/carboplatin and pemetrexed/paclitaxel/docetaxel/gemcitabine/vinorelbine, and the patients treated with chemotherapy were checked by computed tomography scan every 2-3 months until progression disease. All patients with EGFR mutation-positive metastatic lung adenocarcinoma were treated with EGFR-TKIs, i.e., gefitinib and/or erlotinib. This study was approved by the Ethics Committee of Hiroshima University Hospital (IRB33) and all participants provided written informed consent.

### DNA preparation and genotyping

Peripheral whole venous blood samples were gathered from all participants and stored at -80°C. Genomic DNA extraction was performed using the phenol-chloroform extraction and ethanol precipitation methods, as previously described [36]. The *AGER* rs2070600 polymorphism (C/T) was genotyped successfully for all patients and healthy controls. Genotypes were determined using a commercially available polymerase chain reaction (PCR) assay. The PCR mixture contained TaqMan SNP Genotyping Assay (C__15867521_20, C___3293837_1_, C___8848033_1_; Life Technologies Corp., Carlsbad, CA, USA), genomic DNA (2.0 g/mL), and TaqMan Fast Universal PCR Master Mix (Life Technologies Corp.). The real-time PCR analysis was conducted using the Applied Biosystems 7500 Fast Real-Time PCR System (Life Technologies Corp.).

### Calculation of the NLR

At the first visit of each patient, baseline neutrophil and lymphocyte cell counts were measured using an automatic blood cell counter, followed by medical examinations; e.g. interview, blood tests, and chest X-ray, which were performed to exclude patients with infectious disease. The NLR was defined as the absolute neutrophil cell count divided by the absolute lymphocyte cell count obtained using peripheral whole venous blood samples.

### Statistical analysis

Genotype frequencies were calculated by direct counting. The associations of genotype distributions with the susceptibility of lung adenocarcinoma were assessed using Pearson’s chi-squared test and Fisher’s exact test.

The results for the 2 groups were compared using the Mann–Whitney *U-*test and Pearson’s chi-squared test. A multivariate stepwise linear regression analysis was conducted to assess the independent effects of the *AGER* rs2070600 polymorphism and baseline characteristics on NLR in patients with metastatic lung adenocarcinoma. The *t*-test and beta coefficients were used to determine the significance of the predictor and the direction of the relationship. ROC curve analysis was conducted to find the optimal value of NLR for predicting 5-year survival in patients with metastatic lung adenocarcinoma. The 5-year mortality and PFS with platinum-based chemotherapy were evaluated using the Kaplan-Meier approach and the log-rank test. A Cox proportional hazards analysis was used to identify significant predictors of 5-year survival. If the correlation was statistically significant, the multivariate Cox proportional hazards model was used to determine whether the variables independently affected 5-year mortality. A *p*-value of <0.05 was considered significant. All data analyses were implemented in JMP statistical software version 11.2.1 (SAS Institute Inc., Cary, NC, USA).

## Abbreviations

CI: confidence interval
EGFR: epidermal growth factor receptor
EML4-ALK: echinoderm microtubule-associated protein-like 4-anaplastic lymphoma kinase
HR: hazard ratio
IL-6: interleukin-6
MCP-1: monocyte chemotactic protein-1
NLR: neutrophil-lymphocyte ratio
NSCLC: non-small cell lung cancer
PCR: polymerase chain reaction
PDGF: platelet derived growth factor
PFS: progression free survival
PS: performance status
RAGE: the receptor for advanced glycation end-product
ROC: receiver operating characteristic
TAM: tumor-associated macrophages
TKI: tyrosine kinase inhibitors
VEGF: vascular endothelial growth factor
